# The PELskin project: part II—investigating the physical coupling between flexible filaments in an oscillating flow 

**DOI:** 10.1007/s11012-016-0525-9

**Published:** 2016-09-27

**Authors:** Alistair Revell, Joseph O’Connor, Abhishek Sarkar, Cuicui Li, Julien Favier, Laura Kamps, Christoph Brücker

**Affiliations:** 1 School of Mechanical, Aerospace and Civil Engineering (MACE), University of Manchester, Manchester, UK; 2 Laboratoire de Mécanique, Modélisation et Procédés Propres (M2P2), CNRS UMR 7340 - Aix Marseille Université, Marseille, France; 3 Institute for Mechanics and Fluid Dynamics, TU Bergakademie, Freiberg, Germany; 4 Department of Mechanical and Aeronautical Engineering, City University, London, UK

**Keywords:** Flexible filament, Flexible canopy, *Monami*

## Abstract

The fluid-structure interaction mechanisms of a coating composed of flexible flaps immersed in a periodically oscillating channel flow is here studied by means of numerical simulation, employing the Euler-Bernoulli equations to account for the flexibility of the structures. A set of passively actuated flaps have previously been demonstrated to deliver favourable aerodynamic impact when attached to a bluff body undergoing periodic vortex shedding. As such, the present configuration is identified to provide a useful test-bed to better understand this mechanism, thought to be linked to experimentally observed travelling waves. Having previously validated and elucidated the flow mechanism in Paper 1 of this series, we hereby undertake a more detailed analysis of spectra obtained for different natural frequency of structures and different configurations, in order to better characterize the mechanisms involved in the organized motion of the structures. Herein, this wave-like behaviour, observed at the tips of flexible structures via interaction with the fluid flow, is characterized by examining the time history of the filaments motion and the corresponding effects on the fluid flow, in terms of dynamics and frequency of the fluid velocity. Results indicate that the wave motion behaviour is associated with the formation of vortices in the gaps between the flaps, which itself are a function of the structural resistance to the cross flow. In addition, formation of vortices upstream of the leading and downstream of the trailing flap is seen, which interact with the formation of the shear-layer on top of the row. This leads to a phase shift in the wave-type motion along the row that resembles the observation in the cylinder case.

## Introduction

The interaction between an ensemble of flexible slender structures and a surrounding oscillating flow is a generic topic of research encountered in many domains of science, which can be divided in two large categories of coupled problems.

The first one concerns one-way coupled problems, *i.e.* when the motion of the structures is imposed and the fluid flow does not influence this motion. In this category, we find many biomedical applications which involve for instance the motion of beating cilia to transport fluid or mucus in airways [13], cerebrospinal fluid in brain [23], or configurations involving a large number of tasks in human body [14]. Indeed, cilia are whip-like appendages extending from the surface of many types of cells and are ubiquitous in nature. An illustration of this fact is the beating mechanism used to transport mucus in human airways, which is similar to the one used by the cilia found on the body of aquatic animals, which move in water thanks to the rhythmic movement of cilia [5].

The second category is a two-way coupling approach, *i.e.* when the structures are free to move in the fluid, and both fluid and structures influences each other’s dynamics. Several applications exist as well for the two-way coupling, which is sometimes referred as full fluid-structure interaction. A few examples are studies concerning the influence of wind on canopy [6, 20], the dynamics of aquatic vegetation immersed in unsteady flows [15, 16], or the dynamics of cilia found in nature, which are used for self-cleaning or sensing purposes [17, 24].

Other applications can be found in another scientific areas, such as aerospace engineering for instance. Indeed, recent studies have pointed out the benefits of using a poro-elastic coating on wings to control the boundary layer separation, which can lead to reductions of drag coefficient and increase of lift coefficient [3, 8]. The structure of this coating consists in a layer of densely packed slender elements, inspired originally by birds feathers [8], and is thus similar to the one of a canopy made of flexible plants. This porous coating is able to move and deform according to the fluid flow, and allows one to manipulate the flow by using the fluid-structure interaction.

From a modelling perspective, as resolving all the elements of the coating can often lead to prohibitive CPU costs, one may instead consider representative elementary volumes in which volume forces are derived to model the presence of the inner elements, in a homogenised approach [8, 26]. In this way one is able to significantly reduce the number of points required for the fluid mesh, as there is no longer the need to resolve the pore scale. However, correct reproduction of the macroscale elasticity of the porous coating presents a challenge that as of yet is not fully understood - and thus requires further investigation. To date, the models for this purpose have in the main comprised fairly rudimentary elastic assumptions, based on springs and dampers [8] and in that context, Paper 3 of this series, citepelskin3 is addressing this issue by proposing a new and improved homogenized model.

The present paper forms part of a series of outputs summarising the work in the recent EU funded ‘PELskin’ project^1^, wherein focus was placed on investigating the potential amelioration of aerodynamic performance via a Porous and ELastic (PEL) coating. The objective of this project was to elucidate the potential for passive structures to adapt to the separated flow and reduce form drag by decreasing the intensity and the size of the recirculation region. Further investigation of the physical mechanisms described above requires a general set-up, so as to enable a detailed analysis of the flap behaviour under clearly defined conditions. Such a case is proposed here in form of an oscillating channel flow, where a finite row of flexible flaps are implemented. The selected configuration is simple enough to capture the essential characteristics of the coupled problem, and may also be considered to be quasi two-dimensional. In part 1 of this series an experimental study was undertaken for an oscillating flow over a row of 10 flexible flaps spanning the section and two numerical tools were validated against this data.

In the present paper, to investigate the coupled dynamics of fluid and structure, and to bring more information on the physical mechanism to the experimental results, we study the influence of the variation of the mass ratio of the flaps on the flow topology and on the correlation between fluid and structure dynamics. In particular, we place our focus on the waving behaviour observed at the tips of the flexible elements via interaction with the fluid flow. Similar behaviour has been observed in terrestrial or aquatic canopies many times, and frequency lock-in effects have been identified between the natural frequencies of the plants [20, 21], and the shedding of vortices interacting with the tips of the plants. These vortices are generated by a Kelvin-Helmholtz instability which comes from the velocity difference between the external fluid flow and the inner canopy [6]. The advection of these vortices downstream is then promoting a coherent waving motion observed on the canopy, which can be seen easily on wheat fields in windy conditions for instance. This waving motion is called *Honami* in the case of terrestrial canopies and *Monami* for aquatic canopies.

The objective of this research is to provide a quantitative characterization of this fluid-structure interaction mechanism of waving motion using numerical simulation. The unsteady flow configuration of an oscillating channel flow is well adapted to identify phase lags between flapping filaments, and to characterize their time dependent motion along the flow. The numerical framework is based on the Immersed Boundary method coupled to a flow solver, to treat the moving boundaries on a fixed Cartesian grid. Two fundamentally different fluid solvers were used in the PELskin project, to compare their quality in comparison to the experimental data and judge the proper choice for further investigations of such coupled problems. The first is a finite difference code based on Navier-Stokes equations and the second one is a code employing the lattice Boltzmann method. In this paper, all the results have been obtained using the Lattice Boltzmann solver. The dynamics of the flexible elements is modelled using the Euler-Bernoulli equations, as it is done in [12] and [10]. After presenting the numerical method in the next section, results are discussed at the light of other experimental studies in literature on aquatic and terrestrial canopies. A special focus is placed on the extraction of the fluid structure interaction mechanisms generating the *Monami*/*Honami*. Conclusions to this work will be drawn in Sect. 5.

## Case description

**Fig. 1:**
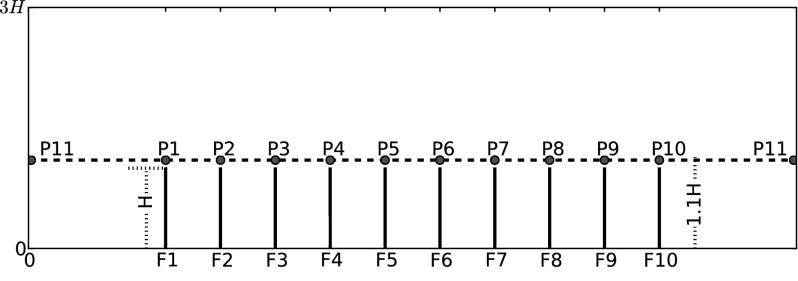
Schematic view of ten identical flexible filaments equally placed at the lower wall of the channel. The letters $F_i$ indicate the position in x-direction of the flexible filament number *i*

An oscillating channel flow of height *L* is generated experimentally in a long tube of square cross-section (6 cm $\times$ 6 cm) over a row of 10 flexible flaps of length *H* at the midpoint, whereby $L=3H$. The flaps are spaced by *H*/2 and are made of silicone rubber (Elastosil RT 601, Wacker Chemie, Germany, Young’s modulus $E = 1.2$ MPa, density $\rho _s = 1.2\,{\text{ g/cm }}^3$), with thickness $d = 1$ mm and span $B = 5$ cm. As such they extend across 83 % of the span of the channel and for the present flow conditions they may be approximated as quasi-2D at the centreline. The flexural rigidity (or bending stiffness) of the flaps is $k = E\times I = 5\times 10^{-6}~{\text {Nm}}^2$ where *I* is the second moment of area along the thin axis of the flap $I = B\times d^3/12$. Figure 1 displays a schematic of the main test section, while the reader is referred to Paper 1 of this series, [7], for complete description of the experimental method and materials.

**Fig. 2:**
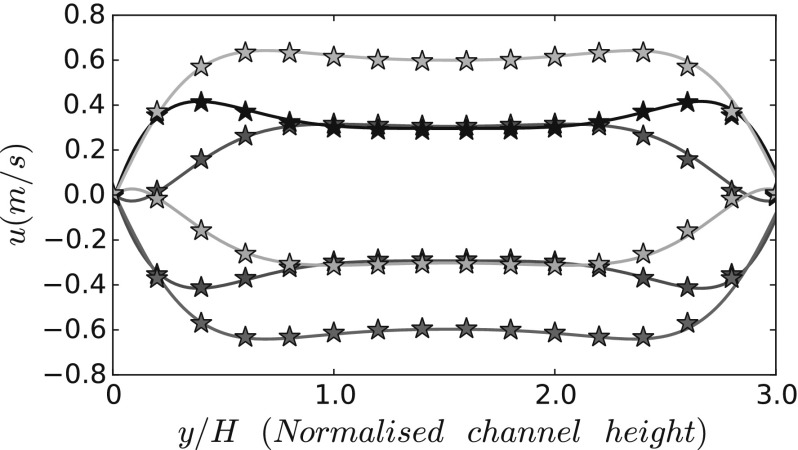
Womersley velocity profiles at inlet position at different instants through one flow oscillation cycle, comparison of analytical solution with numerical results from present work through empty channel. (Color figure online)

The present simulation follows a Womersley velocity profile, defined by analytical expression from [4].

1 $$\begin{aligned} u(y,t)=Ae^{jwt} \left\{ 1-2.0\sum _{n=0}^{\infty }\frac{(-1)^2}{p_n}\left[ \frac{cos(p_ny/b)}{cosh(\gamma _n a/b)}+\frac{cosh(\mu _ny/b)}{cosh(\mu _n)}\right] \right\} \end{aligned}$$

where *A* is the velocity amplitude, $a=b=L/2$, $p_n=((2n+1)/2)\pi$, $\gamma _n=\sqrt{p_n^2+j\eta ^2}$, $\mu _n=\sqrt{q_n^2+j\eta ^2}$, $q_n=((2n+1)/2)\pi (b/a)$ and $\eta =\sqrt{wb^2/\nu }$ refers to the non-dimensional Stokes number, *w* being the angular frequency. Figure 2 provides a good agreement between our simulation results and the analytical solution of [4] at the inlet through one flow oscillation cycle. The Reynolds number of the present simulation is $Re=u_\mathrm{max}H/\nu =120$, based on the maximum stream-wise velocity $u_\mathrm{max}$ and the flexible filament height *H*.

## Method and validation

The lattice Boltzmann method is used to simulate the fluid flow, which is based on microscopic models and mesoscopic kinetic equations; in contrast to Navier-Stokes which is in terms of macro-scale variables. The Boltzmann equation for the distribution function $f=f(\mathbf {x}, \mathbf {e}, t)$ is given as follows:

2 $$\begin{aligned} \frac{\partial f}{\partial t} + {\mathbf {e}} \cdot \nabla _{\mathbf {x}} f + {\mathbf {F}} \cdot \nabla _{\mathbf {e}} f = {\varOmega }_{12}, \end{aligned}$$

where $\mathbf {x}$ are the spatial coordinates, $\mathbf {e}$ is the particle velocity and $\mathbf {F}$ accounts any external force; in the present work this force is the body force $\mathbf {f_{ib}}$ applied to the fluid. Clearly this last term is very important as it will be used to convey the information between the fluid and the structure. The collision operator ${\varOmega }_{12}$ is simplified using the Bhatnagar, Gross, and Krook (BGK) approach [2], where it is assumed that local particle distributions relax to an equilibrium state $f^{(eq)}$ in a single relaxation time $\tau$:

3 $$\begin{aligned} {\varOmega }_{12}= \frac{1}{\tau }\left( f^{(eq)}-f\right) . \end{aligned}$$

This equation is discretised and solved on the lattice, a Cartesian and uniform mesh in our case. At each point on the lattice, each particle is assigned one of a finite number of discrete velocity values. In our case we use the D2Q9 model, which refers to two-dimensional and nine discrete velocities, referred to by subscript *i*. The equilibrium function $f^{(eq)}\left( \mathbf {x}, t\right)$ can be obtained by Taylor series expansion of the Maxwell-Boltzmann equilibrium distribution [22].

Concerning the discrete force distribution needed to keep into account the body force $\mathbf{f_{ib}}$, here we use the formulation proposed by [11], as follows, where *c* is the lattice speed, $c_s=1/\sqrt{3}$ is the speed of sound and $\omega _i$ are the weight coefficients, which take standard values. For further details the reader is referred to [9].

4 $$\begin{aligned} F_i= \left( 1 - \frac{1}{2\tau } \right) \omega _i \left[ \frac{\mathbf {e_i}-\mathbf {u}}{c_s^2} + \frac{\mathbf {e_i} \cdot \mathbf {u}}{c_s^4} \mathbf {e_i} \right] \cdot \mathbf {f_{ib}} \end{aligned}$$

### Immersed boundary method to couple flow solver to structure model

The Immersed Boundary Method (IBM) is used to simulate the moving geometries of the flaps immersed in the unsteady fluid flow. Following this approach, the fluid equations are solved on a fixed Cartesian grid, which do not conform to the body geometry, and the solid wall boundary conditions are satisfied on the body surface by using appropriate volume forces [18, 19]. Following the method proposed by [25] a predicted velocity $\mathbf {u}^*$, computed without the presence of the obstacle, is interpolated ($\mathcal {I}$) onto the embedded geometry of the obstacle, discretized through a number of Lagrangian marker points with coordinates $\mathbf {X}_k$:

5 $$\begin{aligned} \mathbf {U}^* (\mathbf {X}_k,t^n) = \mathcal {I} (\mathbf {u}^*) \end{aligned}$$

Having identified the velocity $\mathbf {U}^*(\mathbf {X}_k,t^n)$ at the location of the Lagrangian markers, a distribution of singular forces that restore the desired velocity $\mathbf {U}^{d}(\mathbf {X}_k,t^n)$ is determined as:

6 $$\begin{aligned} \mathbf {F}^* (\mathbf {X}_k,t^n) = \frac{\mathbf {U}^{d}{(\mathbf {X}_k,t^n)}-\mathbf {U}^* (\mathbf {X}_k,t^n)}{{\Delta }t}. \end{aligned}$$

The singular surface force field is then transformed by a spreading operator $\mathcal {S}$ into a volume force-field defined on the Cartesian mesh points, resulting in the required body force to be used directly in Eq. 4.

7 $$\begin{aligned} \mathbf {f_{ib}} = \mathcal{{S}} \left[ \mathbf {F}^* ({\mathbf {X}}_{k},t^n) \right] . \end{aligned}$$

Where the body surface is in motion, the velocity of each point along the flap is computed via the Euler-Bernoulli equation in non-dimensional form:

8 $$\begin{aligned} \frac{d \mathbf{U^d}^{n+1} }{{dt}} = \frac{\partial }{\partial s}{\left( T \frac{\partial \mathbf {X}_k}{\partial s}\right) } - K_B \frac{\partial ^4 \mathbf {X}_k}{\partial s^4} - \mathbf {F}_{ib} \end{aligned}$$

Here, *T* is the non-dimensional tension of the flap and $K_B$ is the non-dimensional flexural rigidity $k/{K_B}_{ref}$. The reference quantities used for non-dimensionalisation of the equations are: a reference tension $T_{ref}={\Delta }\rho U_0^2$, the reference bending rigidity ${K_B}_{ref}={\Delta }\rho U_0^2 L^2$ and the reference Lagrangian forcing $F_{ref}=\frac{{\Delta }\rho }{L \epsilon \rho _f} U_0^2$. $U_0$ is the characteristic velocity of the fluid flow, ${\Delta }\rho$ is the difference in density per unit area of filament cross section between the filament $\rho _s$ and the fluid $\rho _f$. In the present work we have studied the effect of varying mass ratio; to do this $\rho _f$ is kept constant and the solid density is modified, thereby changing $delta \rho$.

Gravity effects are neglected in the present work.

The closure of Eq. (8) is provided by the inextensibility condition as follows:

9 $$\begin{aligned} \frac{\partial \mathbf {X}_k}{\partial s} \cdot \frac{\partial \mathbf {X}_k}{\partial s} =1 \end{aligned}$$

This condition ensures that the flap length remains constant, and is satisfied using the tension values, which effectively act as Lagrange multipliers. The boundary conditions for the system (8–9) are $\mathbf {X}=\mathbf {X}_0$, $\frac{\partial ^2 \mathbf {X}_k}{\partial s^2} = 0$ for the fixed end, and $T=0$, $\frac{\partial ^2 \mathbf {X}_k}{\partial s^2} = 0$ for the free end. The resulting set of equations are discretised using a staggered arrangement and solved simultaneously using a Newton method, by a direct evaluation of the exact Jacobian matrix, which incorporates the given boundary values.

**Fig. 3:**
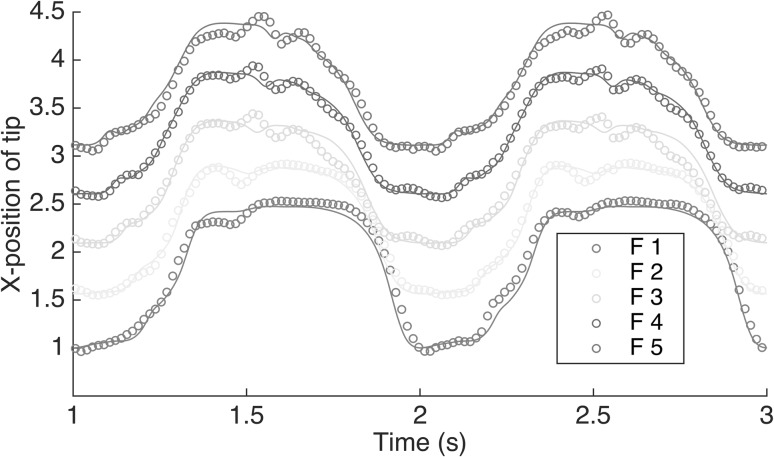
Non-dimensional tip positions of flap in x-direction within three flow cycles (offset by 0.5 for clarity). *Solid curve* numerical results from present work compared to *open circle*: experiment. The letters F*i* indicate the flexible flap number *i*. (Color figure online)

**Fig. 4:**
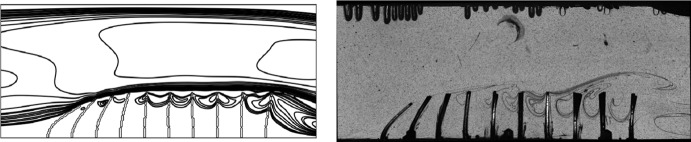
Qualitative comparison of filament dynamics: (*left*) contours of instantaneous flow velocity vectors from numerical simulation; (*right*) Experimental snapshots obtained at the same instant as in the numerical simulation. (Color figure online)

A comprehensive validation was conducted in [7] for a flexible flap without fluid, and then for the two-way fluid structure interaction configuration. The latter case was considered at the same dimensions and same boundary conditions as the experiments. The flexible flaps are mounted on the bottom wall of the channel. The numerical results achieved grid convergence and identified a 2nd order convergence. Figure 3 provides a comparison of flap tip positions in x direction obtained from both flow solvers, the experimental results are also plotted for comparison. Both flow solvers return almost identical results for this case, and comparison to experimental data is also good given the 2D approximation made.

Qualitative validation can be also obtained from the comparison between numerical and experimental results of instantaneous flow velocity vectors, as provided in Fig. 4. These results are discussed in more detail in [7]. During the cycle, the first filament in the array (with respect to the bulk flow direction) starts to deflect from its vertical position before the others, and this deflection is gradually transmitted through the following filaments as the cycle progresses. At the same time, the last filament in the array has also deflected earlier and to a greater extent than its neighbours, as a consequence of the larger recirculation vortex that has by this stage formed aft of the array. In the first stage of this study we have established that the vortex is a primary feature of the flow, since the boundaries between the zones of positive and negative momentum zones are often passing through the vortex core [1, 16]. It is here expected that the size and longevity of this vortex are sensitive to the parameters of the system and this will be investigated in the following sections.

## Variation of the mass ratio

**Fig. 5:**
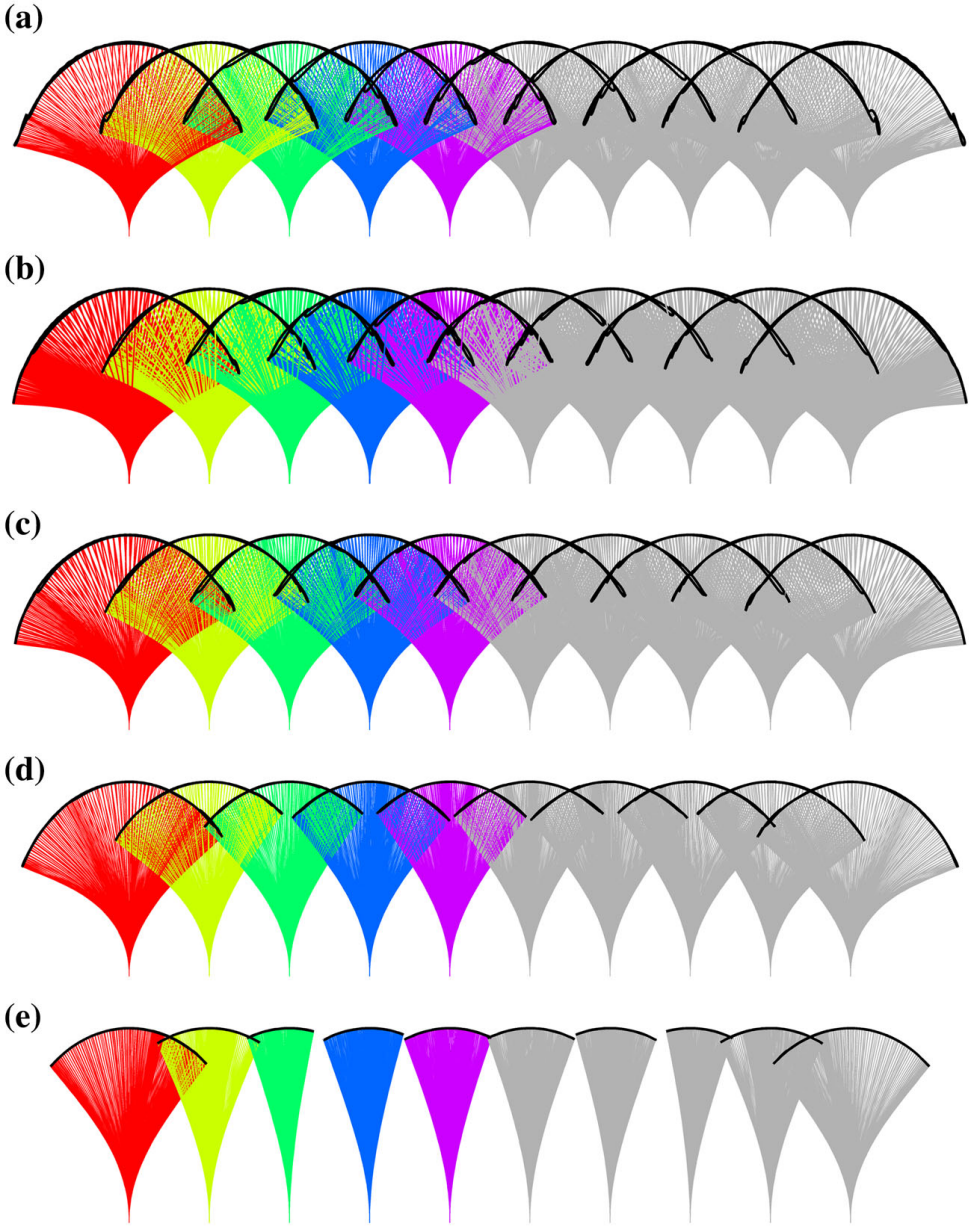
Position diagrams of flap arrays for (*top to btm*:) $\mu ^*=\{0.66,0.8,1.0,1.6,4.0\}$, where flaps $1-5$ are coloured corresponding to legend on Fig. 3. (Color figure online)

In this section we present results where mass-ratio has been varied. With respect to the original configuration from [7], summarised in the previous section, we here evaluate the flow for 4 further values of mass ratio $\mu =\rho _s/\rho _f$, which when normalised by the value from the experimental configuration, form the set $\mu ^*=\{0.66,0.8,1.0,1.6,4.0\}$. In these tests the bending stiffness remains unchanged. The modification of mass ratio directly alters the natural frequency of the flap and as such we expect a modified response from the structure to the imposed flow frequency. Figure 5 displays the overlaid positions of each flap of the array throughout the cycle for the five different cases mentioned previously. These plots provide a clear comparison of how the range of motion and relative interactions of flap vary in each case.

### Flow dynamics

**Fig. 6:**
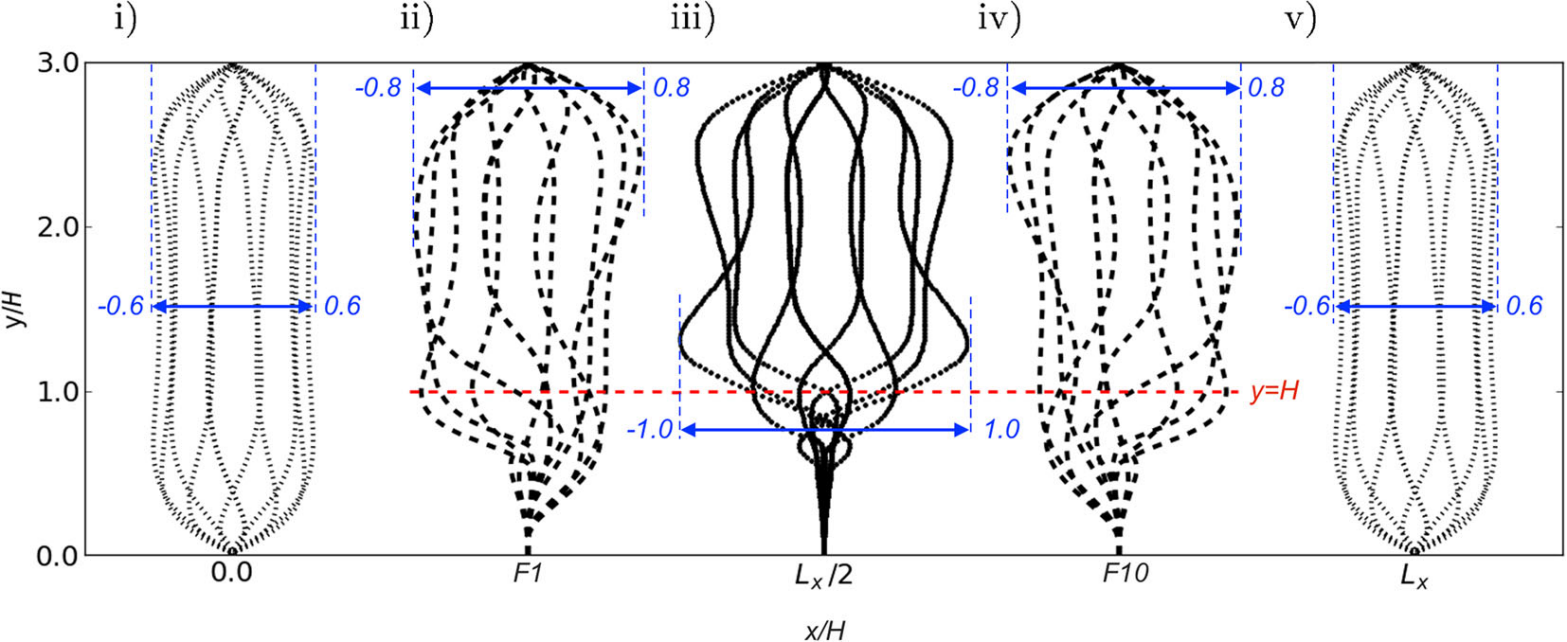
Stream-wise velocity profiles *u* along the channel height at (i) channel entrance; (ii) position of filament 1; (iii) channel center; (iv) position of filament 10; (v) channel exit ($L_x$) through one flow oscillation cycle. The *red line* indicates the filament height and *blue lines* and numbers indicate non-dimensional range of velocity profiles in x-direction. (Color figure online)

The interaction between fluid flow and flexible filaments can be assessed from stream-wise velocity profiles across the channel height, and extracted at five different locations in the x-direction (Fig. 6) for the baseline case. On either sides of the channel, far enough removed from the filament array, the stream-wise flow velocity *u* follows the same Womersley profile as the baseline case in Fig. 2. As can be expected, significant distortions occur on the flow velocity profiles in at the edges of the filament array, i.e. Fig. 6ii, iv. At the centre of the array, Fig. 6 iii, a quasi-Womersley profile is observed above the top of filament layer at the channel center, and a significantly modified velocity profile is exhibited inside the filament layer, indicating a strong influence of the filament motion on the stream-wise flow velocity *u*. The flow at the channel centre is entirely symmetric, as observed in Fig. 6iii. Furthermore, the flow at Filaments 1 and 10 are individually asymmetric while together anti-symmetric, as shown in Fig. 6ii, iii. It is instructive to consider further the flow profiles at the first/last filament location. In particular for the region below the red line indicating the filament height at equilibrium, since in the region $0<y<0.5$ the mean flow over one cycle appears to be directed towards the centre of the filament array.

**Fig. 7:**
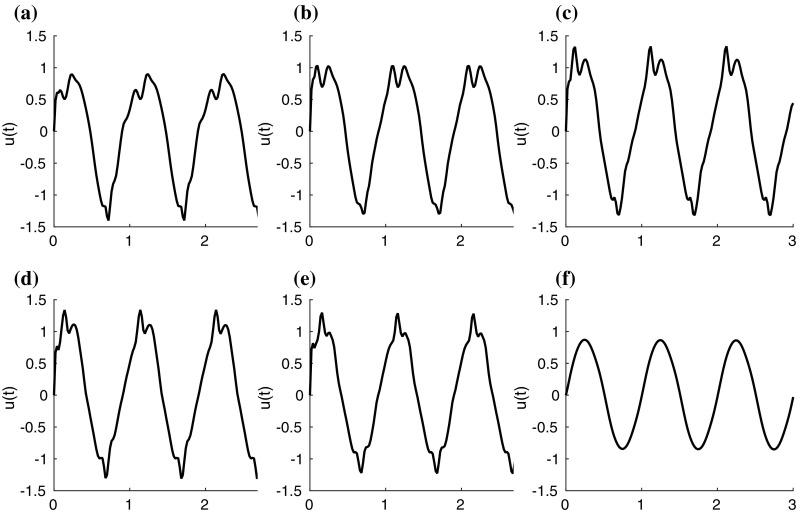
Stream-wise velocity *u* of fluid flow at positions of Fig. 1. **a**–**e** P1–P5 in order; **f** P11

Figure 7 shows the stream-wise velocity *u* within three flow oscillation cycles, corresponding to different positions P1–P5 and P11, at a height of 1.1*H* (see Fig. 1). The magnitude of the stream-wise flow velocity *u* is clearly shown to be larger over the filament top positions P1–P5, with respect to the velocity magnitude at the inlet position P11. A frequency analysis of the stream-wise flow velocity *u* confirms that the dominant frequency peak, of amplitude $A_1$, corresponds to the flow frequency of 1.0Hz. A second peak at about 2.0Hz and of amplitude $A_2$ occurs on the positions P1–P4 which correspond to the extremities of the filament layer. Let $\alpha _{u}=A_2/A_1$ be the spectral ratio of the stream-wise flow velocity *u*, which will be used later on to relate the dynamics of the flow and the filament motion.

**Fig. 8:**
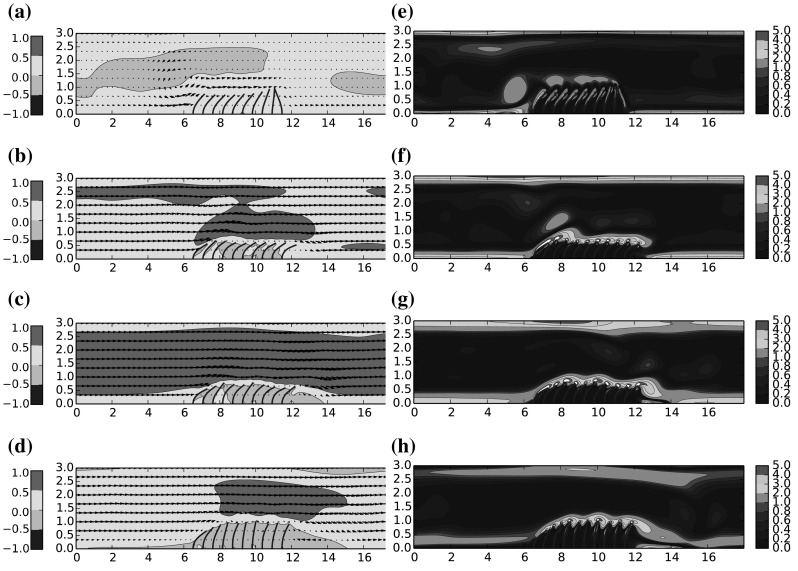
Mass ratio $\mu ^*=0.83$: **a**–**d** Instantaneous flow velocity vectors represented by *arrows* and color contours of stream-wise velocity *u*; **e**–**h** colour contours of instantaneous vorticity. (Color figure online)

**Fig. 9:**
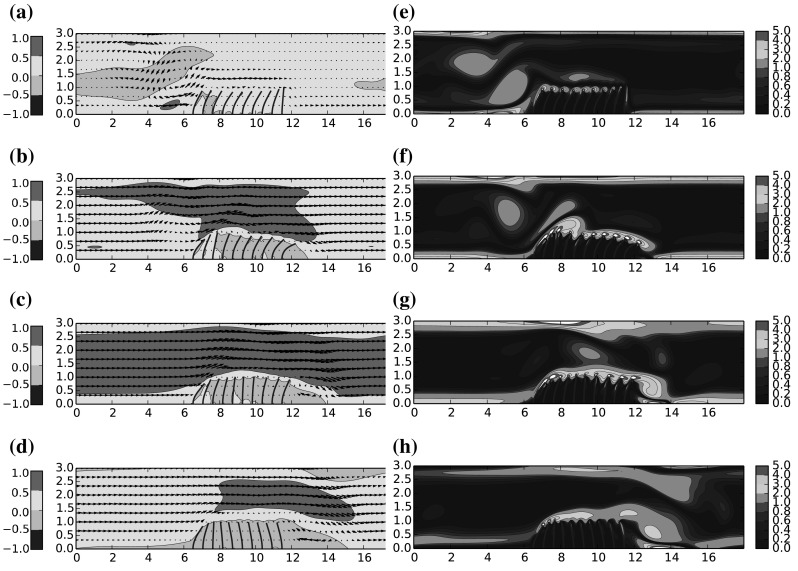
Mass ratio $\mu ^*=1.6$: **a**–**d** Instantaneous flow velocity vectors represented by *arrows* and color contours of stream-wise velocity *u*; **e**–**h** Colour contours of instantaneous vorticity. (Color figure online)

**Fig. 10:**
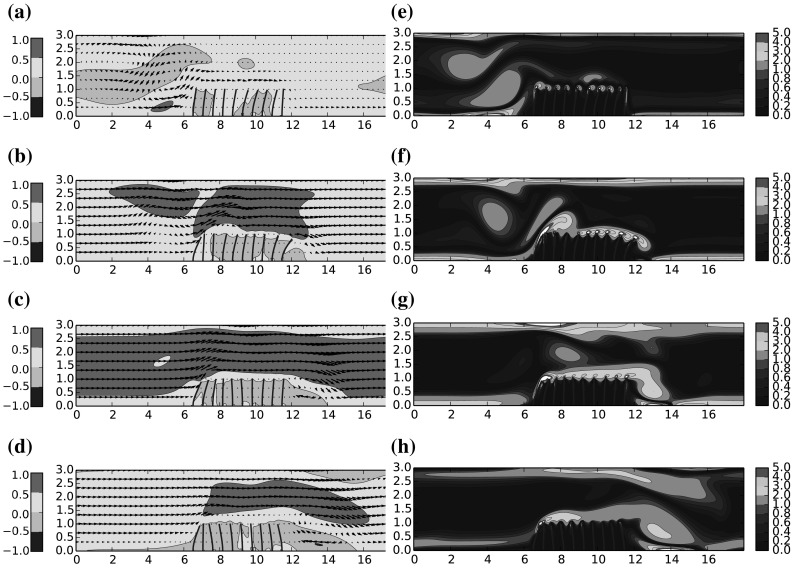
Mass ratio $\mu ^*=4$: **a**–**d** Instantaneous flow velocity vectors represented by *arrows* and color contours of stream-wise velocity *u*; **e**–**h** colour contours of instantaneous vorticity. (Color figure online)

Snapshots of instantaneous velocity field and instantaneous vorticity through one half cycle ($T=0.5$s) of the symmetric flow are given in Figs. 8–10, for $\mu ^*=\{0.66,1.6,4.0\}$ respectively. The results for $\mu ^*=\{0.8, 1.0\}$ were observed to be qualitatively similar to $\mu ^*=0.66$, and are thus omitted for clarity. Under the driving motion of the oscillating flow, the filament motion is significantly influenced by the presence of the vortex which periodically appears near to both sides of the coating and in the following we attempt to elucidate how this influence is modified as a function of $\mu ^*$.

**Fig. 11:**
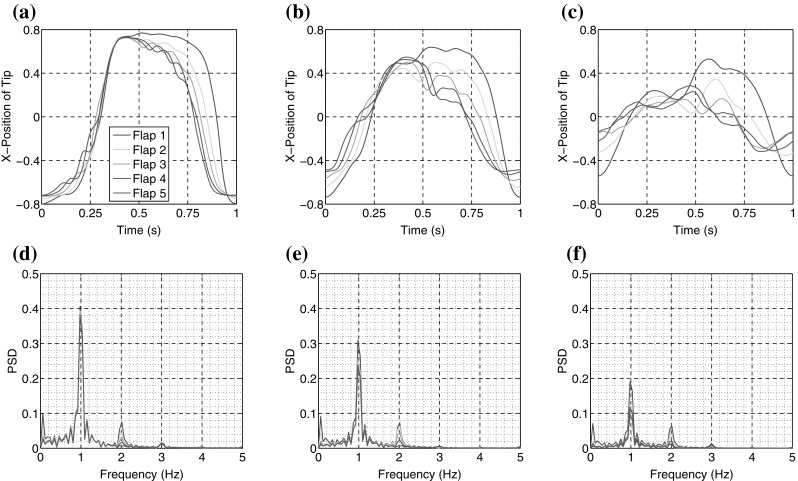
Normalised and superimposed trace of tip deflection (x-component) for flaps 1–5; increasing mass ratio left to right; $\mu ^*=0.66$, $\mu ^*=1.6$ and $\mu ^*=4$. *Bottom row* displays corresponding Fourier transform of trace. (Color figure online)

Considering the plots of instantaneous flow velocity in the first instance, one can identify the effect of increased $\mu ^*$ on the bulk flow through the channel. For low values the bulk flow is able to pass directly over the filament array without significant deflection, while as $\mu ^*$ increases, the filaments yield less to the oncoming momentum and the blockage effect of the filaments in the channel is amplified. The bulk flow is subsequently accelerated and deflected upwards at an angle, which acts to lift the rotational boundary layer away from the tips of the filaments. The main consequence of the previous point is that the shear layer flowing over the filament tips is broadly uninterrupted versus in the higher $\mu ^*$ cases than the lower cases. The facility for an interaction of flow vortices with the natural vibration frequency of the filaments is then removed, and as such there less opportunity for a ‘lock in’ effect between the fluid and the filament array. Indeed the tip deflection profiles in Fig. 11c shows a broadly uncorrelated motion of the filaments compared to Fig. 11a.

Further to the previous points, we observe that for higher $\mu ^*$, the flow recirculation aft of the filament array is more pronounced, and the vorticity in this region is increased. This has two notable effects; first the filaments in the aft location, normally deflected downstream in the direction of the bulk flow, are instead deflected back towards the centre of the filament array. Furthermore, the low pressure region corresponding to the vortex core appears to act to detach the flow at the point on the channel ceiling immediate above this location, deflecting the bulk flow down towards the channel floor. We note here that such a mechanism might be relevant for flow control.

For higher $\mu ^*$, it can be seen that there is no contact between any of the filaments, however for the cases of smallest mass ratios, $\mu ^*=\{0.66,0.8\}$, there are short instances amounting to $\sim 10\,\%$ of the cycle where the final two filaments on the ley side of the array appear to come into contact at their tips, as seen from Figure a. We do not presently include any interaction model in these cases and as such there is nothing to prevent this from occurring. Encouragingly there does not seem to be an adverse numerical behaviour resulting from this contact—the filaments are naturally able to fully separate following these moments of close proximity. Since the filament positions are computed independently from one another, one filament has no direct awareness of another’s location, and as such they do not directly ‘touch’; but they do occupy the same hydrodynamic field, and in regions of low velocity both will be momentarily stationary. For cases where such contact is frequent and substantial we would need to incorporate collision modelling to correctly handle contact and lubrication effects; but since in the present calculations these instances are rare it is not expected to have significant bearing on our current conclusions.

### Flap dynamics

Figure 11 displays the normalised and superimposed trace of tip deflection (x-component) for mass ratios $\mu ^*=\{0.66,1.6,4.0\}$. Only flaps 1–5 are included since flaps 6–10 are antisymmetric to this. Also included in the figure are the corresponding Fourier transform of trace, where 1 Hz corresponds to forcing frequency of the case. Since all plots are scaled to the same values, comparisons between amplitudes can be drawn directly. The results indicate that for low mass ratios, the flap motion is relatively coherent, with all flaps covering a similar range of x-coordinate. There is a phase lag present as discussed in the previous section, but this is minor compared to the results which follows. For $\mu ^*=1.6$, there is a drop in range of motion, as indicated by a reduced range of x-coordinate. There is also a marked drop in coherence, with a significant spread of motion over the second half of the cycle. This may be associated with a sharper ‘kick’ of the flap tips in these cases. For $\mu ^*=4$ the trend continues and flaps are without a clear coherent motion over the entire cycle. Considering the main peak in the frequency plots, at 1Hz, similar conclusions are made, where coherence is now represented by amplitude of the key frequencies. For $\mu ^*=0.66$ the coherence is strong, and this decreases notably for each subsequent increase in $\mu ^*$.

**Fig. 12:**
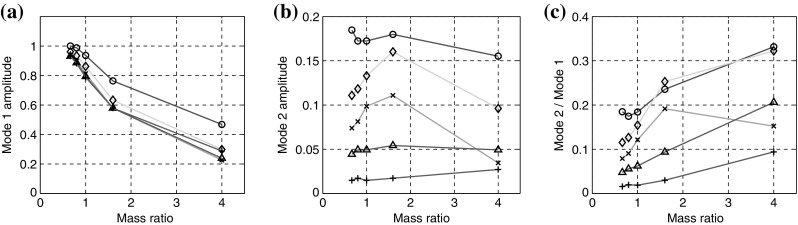
Normalised amplitude of flap motion against normalised mass ratio $\mu ^*$, for (*left*) amplitude $B_1$ of primary frequency; (*middle*) amplitude $B_2$ of first harmonic; (*ratio*) ratio of amplitudes $B_2/B_1$. (Color figure online)

Further insight can be gained from Fig. 12, which plots the normalised amplitude of flap motion against normalised mass ratio, for each of the flaps 1–5, (see previous figure for legend). Three plots are included; the driving frequency of $f_1=1$ Hz, the first harmonic of $f_2=2$ Hz and the ratio $f_2/f_1$. From the first plot it becomes clear that the energy at $f_1$ decreases with $\mu ^*$, rapidly at first then more slowly, following an asymptotic variation. Consistently the motion response of the first flap, is around 25 % higher than flaps 2–5, which are remarkably similar across the range of $\mu ^*$ tested. In contrast, the corresponding results for $f_2$ demonstrate a broader variation for each of the flaps 1–5. For the lowest value of mass ratio, the amplitude of $f_2$ varies almost linearly from flap 1 to flap 5, as might be expected. Indeed, flaps 1, 4 and 5 indicate almost constant values of amplitude over the range of mass ratios tested. In stark contrast, flaps 2 and 3 rise with mass ratio, reaching a peak at the tested value of $\mu ^*=1.6$, before again reducing for $\mu ^*=4$. This peak in amplitude can be related to the kick mentioned previously, and there appears to be a kind of increased sensitivity to $\mu ^*$ for flaps 2 and 3, compared to the other flaps. It appears that this dynamic excitation results directly from the large coherent vortex that passes over these flaps. Also of note, in the case of $\mu ^*=4$, the 3rd flap moves less than the 4th, as indicated also in Fig. 5. The final plot of Fig. 12 displays the ratio of the amplitudes of the frequencies, here defined $\alpha _{f}=B_2/B_1$, which indicates that as mass ratio increases, more energy is manifested in the higher frequency range. This is confirmed by the increased amplitude of higher frequency oscillations reported in Fig. 11.

### Spectral analysis

In this section we attempt to correlate the dynamics of the flaps with the dynamics of the fluid in their immediate vicinity. In the previous sections we have quantified the dynamic response of filament motion and fluid motion as $\alpha _{f}$ and $\alpha _{u}$ Respectively. Further we here quantify the asymmetry of the filament motion by introducing a parameter which measures the ratio of time spend by the filament tip on the right hand side of it’s base, ${\Delta }t_{1}$, versus the time on the left hand side ${\Delta }t_{2}$; i.e. summarised as ${\Delta }t=\Vert {\Delta }t_{2}-{\Delta }t_{1}\Vert$, and normalised by flow oscillation period, *T*. Clearly for a symmetric motion this value will be zero.

**Fig. 13:**
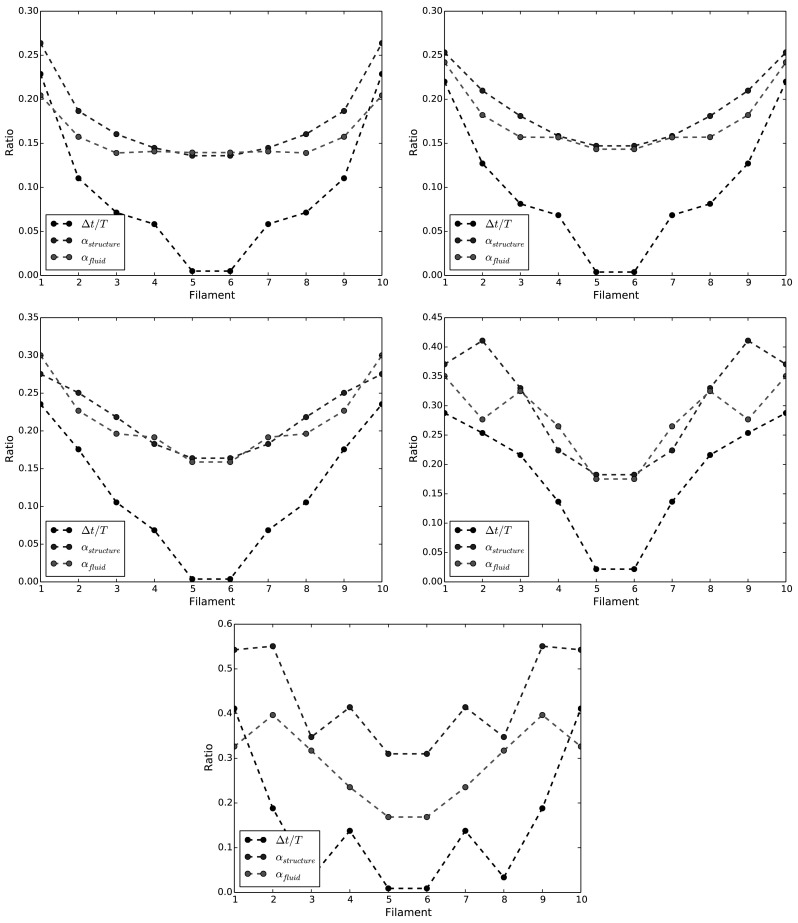
Spectral ratios for the fluid ($\alpha _{u}$) and the filaments ($\alpha _{f}$), together with phase lags ${\Delta }t/T$ at positions P1–P10, for a range of mass ration $\mu ^*=\{0.66,0.8,1.0,1.6,4.0\}$. The x-axis indicates the filament positions in x-direction. (Color figure online)

Figure 13 displays the variation of $\alpha _{f}$, $\alpha _{u}$ and ${\Delta }t/T$ for the set of parameters tested. The values of all three ratios follow the same trend moving towards the side of the layer in almost all cases. This demonstrates that the frequency content of the stream-wise velocity of the flow in the rods region is significantly modified by the motion of flexible rods, which underlines the fact that the layer of flexible flaps does not behave as a passive vibrating medium in the fluid. For $\mu ^*=\{0.66,0.8,1.0\}$, the two quantities $\alpha _{f}$, $\alpha _{u}$ are surprisingly close, particularly so for the baseline case, intended to display ‘lock-in’ characteristics by the experimenters. In all cases, the quantity ${\Delta }t/T$ reaches zero for the central pair of filaments; indicating that an entirely symmetric filament motion is achieved at this point.

For values of mass ratio corresponding to $\mu ^*=1.6$, the filaments are found to beat at the same frequency of the fluid in the center of channel. A loss of correlation between the fluid and the structure dynamics is observed on the sides, as these zones are more dominated by the presence of a strong coherent vortex. This is confirmed upon comparison of Figs. 8g and 9g. The particular loss of correlation which occurs at the second filament, is also identified in Fig. 12b where there appears to be an amplification mechanism in play for this particular mass ratio.

When the mass ratio is increased further to $\mu ^*=4$, the correlation is found to be even weaker, leading to a quasi-uncorrelated dynamics for fluid and structure. In this case, the vortices created on the sides of the layer are advected upwards, and thus influence much less the motion of the flaps, as they remain in a flow region too far away from the flaps. This can be assessed clearly on Fig. 10g for instance, where it can be seen that the vorticity is transported more towards the top of the domain compared to Fig. 8g, where the vorticity is transmitted to the flaps and thus leads to a more correlated dynamics between the fluid and the flaps.

## Conclusions

The coupling mechanisms involved in the two-way interaction between an incompressible oscillating channel flow and a coating made of flexible filaments have been investigated in the present work. A quantitative characterization of these physical mechanisms has been provided. From the present results, the phase difference of adjacent filaments, which leads to the *Monami* or *Honami* waving motions, has been characterized by means of several analysis, which have led to the following observations.

From the detection of coherent eddies, it is demonstrated that the flow vortex, which periodically occurs near the filament coating sides, is the cause leading to the smoothly varying phase difference of adjacent filaments, and is related to large vorticity. The effect of the flexible filaments on flow dynamics has been highlighted from the FFT spectra results. Over the flexible filament top region, the spectral ratio $\alpha _{u}$ of the stream-wise flow velocity is found to be quantitatively close to the phase difference ratio ${\Delta }t/T$ and the spectral ratio $\alpha _{f}$ of the filaments, demonstrating that the layer of flexible flaps does not behave as a passive vibrating medium in the fluid.

Over the course of the cycle there is a mean flow from the outer filaments towards the centre, which is characterised by the measure of asymmetry, demonstrating that the first and last filaments spend more time deflected ‘into’ the array than away from it. This hysteresis effect demonstrates the poro-elastic nature of the filament array. Under tuned conditions, the spectral responses of the filament and the adjacent fluid are closely linked, though at a certain threshold the coupled motion breaks down as the dynamic response of the structure grows faster than that of the fluid and the correlation breaks down.

At high mass ratios, the structural response is further uncoupled from the flow, which is deflected beyond the tips of the filaments, further reducing the potential flow-filament interaction and thus the coupling. Having demonstrated a relatively narrow band of the parameter space under which dynamics are closely coupled, a significant departure from these conditions is observed for relatively small change of input parameter; the implications for flow control via a poro-elastic layer are significant and further work is under way to link these findings to the unsteady flow field in the wake of a bluff body.

## Footnotes

1
http://www.transport-research.info/project/pel-skin-novel-kind-surface-coatings-aeronautics.
